# Macrophages in solid organ transplantation

**DOI:** 10.1186/2045-824X-6-5

**Published:** 2014-03-11

**Authors:** Xinguo Jiang, Wen Tian, Yon K Sung, Jin Qian, Mark R Nicolls

**Affiliations:** 1 Department of Medicine, VA Palo Alto Health Care System/Division of Pulmonary/Critical Care, Stanford University School of Medicine, Stanford, CA 94304, USA; 2 VA Palo Alto Health Care System, Bldg 101, A4-151, 3801 Miranda Ave, Palo Alto, CA 94304, USA; 3 VA Palo Alto Health Care System, Med111P, 3801 Miranda Ave, Palo Alto, CA 94304, USA

**Keywords:** Macrophage, Transplantation, Ischemia reperfusion injury, Acute rejection, Graft vascular disease

## Abstract

Macrophages are highly plastic hematopoietic cells with diversified functions related to their anatomic location and differentiation states. A number of recent studies have examined the role of macrophages in solid organ transplantation. These studies show that macrophages can induce allograft injury but, conversely, can also promote tissue repair in ischemia-reperfusion injury and acute rejection. Therapeutic strategies that target macrophages to improve outcomes in solid organ transplant recipients are being examined in preclinical and clinical models. In this review, we discuss the role of macrophages in different types of injury and rejection, with a focus on macrophage-mediated tissue injury, specifically vascular injury, repair and remodeling. We also discuss emerging macrophage-centered therapeutic opportunities in solid organ transplantation.

## Introduction

Macrophages are ancient cells in metazoan phylogeny that play critical roles in detecting and eliminating harmful pathogens. They were first identified and described more than a century ago by Élie Metchnikoff [1] and are an essential component of the innate immune system, forming the first line of defense against infectious agents [2,3]. In response to pathogens, macrophages accumulate in tissues both through the recruitment and differentiation of circulating monocytes as well as through in situ proliferation [4,5]. There, they bind to Toll-like receptor (TLR) ligands such as lipopolysaccharide (LPS) or interferon-γ (IFN)-γ and are induced into an activation state that is characterized by a shift from aerobic metabolism to anaerobic glycolysis, increased production of proinflammatory mediators, augmented expression of inducible nitric oxide synthase (iNOS) and synthesis of reactive oxygen (ROS) and nitrogen species (RNS). This phenotype leads to efficient digestion of engulfed pathogens and is known as the classically-activated macrophage (CAM) [3,6]. In addition to their participation in host defense, macrophages have also been shown to play key roles in a range of physiological processes, including development, homeostasis, tissue repair, as well as pathologic processes including fibrosis, obesity and malignancy. These macrophages are induced by exposure to interleukin (IL)-4 and IL-13 and have a phenotype that is different from the CAMs. They are classified as alternatively-activated macrophages (AAMs) [2,3,6-9]. Finally, the regulatory macrophage (Mreg) has been recognized for its anti-inflammatory properties [10] and may play a protective role in transplant recipients.

CAMs and AAMs are routinely classified as ‘M1’ and ‘M2’ respectively [9]. However, they display tremendous heterogeneity, changing their phenotypes dramatically in response to cues from the microenvironment. To address these different phenotypes, M2-polarized macrophages have been sub-classified into M2a, M2b and M2c to discriminate their differentiation status in recent years [11]. In reality, even this more recent classification scheme does not clearly represent the very wide array of macrophage types manifesting highly diverse functions and phenotypes. While a more detailed classification of macrophages based on gene expression profiles or differentiation status will facilitate target identification for therapeutic interventions in various pathological conditions [2,9], the fact that macrophages are so highly mutable is a persistent consideration in scientific explorations of these cells. A recent landmark study evaluating the transcriptome of human macrophages induced by a variety of stimuli revealed an extraordinary spectrum of macrophage activation states that far extend the current M1 versus M2-polarization model [12]. For these reasons, this review generally avoids the M classification scheme and focuses instead on macrophage phenotype and function.

It has been recognized since the 1970s that macrophages are involved in the rejection of solid organ transplants [13,14]. Macrophages have been shown to play a role in cell- and antibody-mediated rejection as well as in the development of graft vascular disease (GVD), a manifestation of chronic rejection [15,16]. Macrophages may promote the development of acute rejection by producing ROS, eicosanoids and cytokines such as IL-1, tumor necrosis factor (TNF)-α and IL-18 [17,18]. On the other hand, macrophages may also dampen the alloimmune response by acquiring a regulatory phenotype that has been recently described [18]. Lastly, monocytes/macrophages may also help repair injured allograft microvasculature by producing proangiogenic factors [19].

Currently, immunosuppressive therapy regimens in organ transplantation primarily target T cells. As transplant outcomes continue to be suboptimal [20,21], identification and characterization of macrophages with distinct phenotypes may provide novel therapeutic targets to improve transplant survival. In this review, we will highlight studies that provide new insights into the role that macrophage play in different types of allograft injury and rejection and conclude with potential therapeutic strategies to promote allograft health.

## Macrophages in ischemia reperfusion injury (IRI)

Ischemia – reperfusion describes the condition of an organ during the procurement and transplantation process. When an organ is harvested, the blood flow to the organ is cut off and cooled with physiologically-buffered solution - a state known as cold ischemia. Reperfusion occurs after the organ is transplanted and blood perfusion and oxygenation is restored. Reperfusion exacerbates the initial ischemia-induced tissue injury by triggering adaptive and innate immune responses [22] including macrophages (as described in greater detail below). The pathophysiological features of IRI include: 1) impaired endothelial barrier function with increased vascular permeability and leakage, 2) promotion of donor cell death programs, including apoptosis, autophagy-induced cell death and necrosis, 3) transcriptional reprogramming of donor cells, by upregulation of hypoxia inducible factor (HIF) and nuclear factor κB (NF-κB)- induced gene expression, 4) activation of the complement system and 5) activation of TLRs on macrophages and donor parenchymal cells [22-25].

IRI of the transplanted organ has long been recognized as a non-allogeneic factor that influences graft function and survival [26] and macrophages are key components of this pathology [22]. Macrophages have been shown to accumulate during the early phase of IRI in kidney and liver transplants [27,28]. Recently, it was shown that inhibition of sphingosine kinase-2 (SK-2) led to decreased macrophage accumulation in liver transplants, an effect that correlated with attenuated graft IRI [29]. In cardiac transplantation, decreased infiltration of macrophages during IRI also correlated with improved microvascular health and graft survival [30]. Also, alveolar macrophages are thought to be essential in the initiation of IRI in lung transplantation. They have been shown to induce tissue injury through the production of ROS and proinflammatory cytokines including IL-8, IL-12, IL-18, TNF-α and platelet activating factor (PAF) [31]. Furthermore, it has been demonstrated that increased macrophage recruitment into rat lung allografts induced by IRI is associated with impaired endothelial cell (EC) barrier function, and EC injury can be diminished when macrophage infiltration is decreased [32]. These studies provide compelling evidence that macrophages play a deleterious role in IRI and contribute to microvascular EC injury following transplantation. Moreover, macrophages accumulated during IRI may also help boost and maintain the adaptive T cell response by producing proinflammatory mediators and by acting as antigen presenting cells [33].

TLR signaling has been shown to be essential for macrophage activation [34]. In a study of spinal cord injury, it was shown that TLR4 deficiency protected the spinal cord from IRI in mice. This study went on to show that hypoxia and deprivation of glucose induced TLR4 expression on macrophages and that TLR4-deficient macrophages produced much lower levels of TNF-α and IL-6 [35]. These findings suggest a mechanism for TLR4-dependent macrophage-induced IRI. In a cardiac IRI study, TLR4-dependent, high-mobility group box-1 (HMGB-1)-activated macrophages produce IL-23, which in turn induced IL-17 production and caused heart allograft injury [36]. This result provides a mechanistic link between macrophages and IL-17-mediated IRI. In brain IRI, peroxiredoxin family proteins were also shown to induce IL-23 production in macrophages through activation of TLR2 and TLR4 signaling [37]. These recent studies from both the transplant and non-transplant animal models further demonstrate that activation of macrophage TLRs is required for macrophage-induced IRI and suggest that TLR-mediated macrophage activation likely contributes to IRI in newly-transplanted organs.

While macrophages have been shown to be harmful and cause allograft injury, they have also been shown to play a reparative role in both transplant and non-transplant IRI. In kidney, a macrophage-specific deletion of wnt7b significantly hindered tissue repair and regeneration following IRI [38]; this study suggests that wnt7b may also play a protective role in organ transplantation. In another study of acute renal injury and repair, it was shown that colony stimulating factor-1 (CSF-1), a hematopoietic growth factor, promotes tissue repair by enhancing tubular cell proliferation and diminishing its apoptosis and that this effect partially depends on the function of macrophages [39]. In lung allografts, polarization of macrophages by prednisone preconditioning diminished IRI and prolonged graft survival [40], suggesting that macrophages with an anti-inflammatory phenotype may also be beneficial during the late stage of IRI. Another recent study showed that treatment with human umbilical cord-derived stromal cells reduces renal IRI and that the beneficial effect depends not only on the presence of macrophages, but also on polarization in the later repair phase [41]. This result further supports the notion that in contrast to the deleterious effect of infiltrating macrophages seen in early IRI (1-3 days post transplant), Macrophages may act in a reparative role in late IRI (3-5 days) [42]. The leukotriene B_4_ receptor type-1 (BLT1) was recently shown to facilitate macrophage recruitment to the IR injured liver and BLT1 deficiency leads to decreased EGF production and impaired tissue repair [43], suggesting a role of macrophage-produced growth factor in tissue regeneration. These studies collectively demonstrate that phenotypically-distinct macrophages exist in different IRI phases and differential targeting strategies, such as depletion or phenotypic polarization, are needed to harness the macrophage as a therapeutic target to prevent or attenuate IRI in solid organ transplants.

## Macrophages in acute allograft rejection

Acute rejection (AR) is a result of the alloimmune attack against the graft and is characterized by an inflammatory pathology that is generally reversible with early immunosuppressive intervention [33]. EC injury and vascular damage is a well-known phenomenon in AR [44,45] and macrophages are increasingly appreciated as an important player in both cellular and antibody-mediated AR [16]. Here we first highlight recent advances in macrophage biology in the setting of solid organ transplantation and then discuss in detail how the ECs of the allograft microvasculature can be damaged as well as repaired by different types of macrophages during AR.

In a clinical study, CD68^+^ macrophages but not T cell infiltration was shown to be associated with renal allograft dysfunction during AR [46]. Consistent with this finding, a pre-clinical study of kidney transplantation showed that macrophage depletion with liposomal-clodronate significantly attenuated graft damage during AR [47]. More recently, inhibition of Rho kinase was demonstrated to promote allograft function and this beneficial effect was associated with decreased macrophage infiltration in renal transplants [48]. Additionally, intravascular macrophage accumulation has been observed in cardiac allografts undergoing antibody-mediated rejection [49]. Furthermore, the accumulation of intravascular macrophages in early human cardiac transplantation also predicts the presence of donor specific antibodies (DSA), C4d deposition and symptoms of antibody-mediated rejection [50]. Collectively, these studies suggest that macrophages play an important role in both cellular and antibody-mediated rejection.

As stated above, macrophages accumulate within a tissue by recruitment of monocytes from the circulation and through proliferation of resident cells. The mechanisms associated with macrophage accumulation in solid organ transplants have been extensively studied. CD99 expressed on ECs is required for monocyte migration through EC junctions [51] and EC expression of P-selectin is also needed for macrophage accumulation in cardiac allografts during antibody-mediated rejection [52], suggesting that EC expression of adhesion molecules is required for monocyte/macrophage extravasation and subsequent tissue accumulation. Chemokines such as monocyte chemoattractant protein-1 (MCP-1), macrophage colony-stimulating factor (M-CSF, also known as CSF-1) and macrophage migration inhibitory factor (MIF) were shown to be positively associated with the number of infiltrated CD68^+^ or ED1^+^ macrophages in renal allografts [53-56]; additionally, chemokine receptors such as CX3C chemokine receptor 1 (CX3CR1), CC chemokine receptor 5 (CCR5) and CXC chemokine receptor 3 (CXCR3) have also been shown to mediate macrophage recruitment in renal grafts [57,58]. Interestingly, while the RANTES/CCR5 pathway also contributes to macrophage accumulation in cardiac transplants, CCR5 blockade only modestly prolonged allograft survival likely because the recruitment of regulatory T cells also requires this signaling pathway [59,60]. These studies collectively demonstrate that multiple chemokines are involved in the recruitment of macrophages into allografts during AR and blocking chemokine-induced signaling pathways may be a promising therapeutic strategy. Other studies have shown that local macrophages proliferate in AR [55,56,61], suggesting another mechanism for increasing the number of macrophages in allografts undergoing AR.

Once in the allograft, macrophages have been shown to promote inflammation, induce tissue damage and secrete inflammatory mediators. CAMs have been shown to produce ROS and RNS, which are probably the primary mediators of tissue damage in AR [47,62-67]. Cytokines such as IL-1β, IL-12, IL-18, TNF-α and IFN-γ have also been shown to be secreted by macrophages. These cytokines not only activate ECs and promote cytotoxic T cell generation, but also induce the production of chemokines such as CSF-1 and MCP-1 [68].

**Figure 1:**
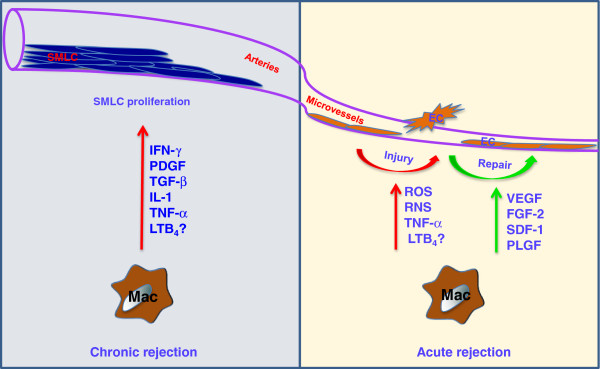
**Macrophages and graft vasculature.** During acute rejection, macrophages induce microvascular EC injury through the production of ROS, RNS, TNF-α and possibly LTB_4_. On the other hand, macrophages can also promote microvascular repair through the production of angiogenic factors, such as VEGF, FGF-2, SDF-1 and PLGF. During chronic rejection, macrophages promote SMLC proliferation by producing IFN-γ, PDGF, TNF-α, IL-1, TGF-β and possibly LTB_4_. Abbreviations: IFN, interferon; ROS, reactive oxygen species; RNS, reactive nitrogen species; TNF, tumor necrosis factor; PDGF, platelet-derived growth factor; IL, interleukin; TGF, transforming growth factor; LTB_4_, leukotriene B_4_; SMLCs, smooth muscle-like cells, VEGF, vascular endothelial growth factor; FGF, fibroblast growth factor; SDF, stromal cell-derived factor; PLGF, placental growth factor.

To explain why macrophage-mediated microvascular EC injury is relevant to transplantation, it is useful to consider several lines of evidence demonstrating the relevance of microvascular health in allograft function and why macrophage injury to the microvasculature may negatively affect the transplant. In a mouse orthotopic tracheal transplantation model, our group has shown that EC death and loss of the graft microvasculature during AR precedes (and is likely a key driver for) the development of airway fibrosis (i.e. chronic rejection) [69]. We subsequently demonstrated that the activation of the complement system and CD4^+^ T cells (but not CD8^+^ T cells) independently induces airway microvascular loss following transplantation [70]. We also showed that donor cell-expressed HIF-1α is associated with airway microvascular health and augmented expression of HIF-1α using adenovirus-mediated gene transfer prolongs EC survival, promotes vascular repair, and results in delayed and attenuated airway fibrosis [19]. These data are consistent with clinical studies which show that microvascular loss precedes and, consequently, may play a causal role in chronic rejection [71-74]. This concept emphasizes the importance of understanding how microvascular ECs are injured and developing new therapeutic targets to protect them during AR. Macrophages have been shown to induce EC apoptosis through activation of the Wnt pathway in patterning the eye vasculature during development [75]. Macrophages can also induce EC death through iNOS-derived nitric oxide [76]. We recently demonstrated that the lipid mediator leukotriene B_4_ (LTB_4_) produced by macrophages in pulmonary hypertension lungs induced EC apoptosis; LTB_4_ was found to induce significant EC apoptotic death in a dose-dependent manner within 24 hours of culture [77]. By extension, it is possible that macrophage- produced LTB_4_ may also induce allograft EC apoptosis during AR. Thus, macrophages may directly or indirectly induce EC death through production of cytotoxic molecules or proinflammatory mediators during AR (Figure 1).

Despite the injurious effects on ECs, macrophages have also been shown to promote vessel growth in tumors [78-80] and angiogenesis in the hind limb ischemia model [81]. These macrophages are identified by expression of the Tie2 receptor. In the airway transplantation model, our group has also shown that Tie2-expressing monocytes/macrophages contribute to donor microvascular repair during AR [19]. Tie2-expressing monocytes/macrophage may promote graft microvascular repair by producing proangiogenic growth factors such as vascular endothelial cell growth factor (VEGF), placental growth factor (PLGF), stromal cell-derived factor (SDF)-1 and fibroblast growth factor (FGF)-2 [19,82]. In addition, increased expression of HIF through Von Hippel–Lindau (VHL) knockdown in recipient derived Tie2 lineage cells promotes donor vascular regeneration and limits graft invasion by aspergillus [83]. These studies suggest that during AR, a subpopulation of macrophages may help repair the injured graft microvasculature and therefore, deeper characterization of macrophages in AR is needed for efficient therapeutic targeting. Figure 1 demonstrates the myriad of effects that graft-infiltrating macrophages may have on donor microvessels and arteries.

In addition to graft-protective functions likely conferred by angiogenic macrophages, macrophages with regulatory function capable of quelling maladaptive inflammation likely serve a protective role in transplantation. Research in the last decade has identified numerous mechanisms that can induce Mregs both *in vitro* and in preclinical animal models [11], including macrophage stimulation by M-CSF, IL-10, vitamin D3, glucocorticoids and prostaglandin E2 [84-88] as well as macrophage repetitive stimulation by TLRs [89,90]. The human Mreg has also recently been generated by culturing CD14^+^ peripheral blood monocytes for 7 days in the presence of M-CSF and 10% human serum plus a 24-hour IFN-γ pulse [91]. These Mregs have been shown to be able to potently suppress T cell proliferation through IFN-γ-induced indoleaminepyrrole 2,3-dioxygenase (IDO) production and contact-dependent depletion of activated T cells [92]. In addition, a recent in depth phenotypic and functional characterization of the mouse Mregs revealed that these cells belong to a subset of suppressor macrophages expressing markers that distinguish them from the M1- and M2-polarized states [93]. In vitro, these Mregs completely suppress polyclonal T cell proliferation in an iNOS-dependent and allospecific fashion and administration of *in vitro*-derived Mregs significantly reduces acute rejection and prolongs the survival of the mouse cardiac allografts [93]. This study suggested that macrophages may also protect the vascular EC by differentiating into a regulatory subtype and consequently suppressing alloreactive T cells. This study also demonstrated that Mregs may be produced *in vitro* and could potentially be used as a source of cellular therapy for tolerance induction with a reduced dosage of immunosuppressive drugs in solid organ transplantation.

## Macrophages in GVD

Chronic rejection is the leading cause of graft rejection, which is manifested by transplant tissue fibrosis and/or GVD [15,19,33]. GVD is the single most important limitation to long-term survival of transplanted solid organs [15]. It is traditionally seen in the arterioles and the arteries and may affect the entire length of the arterial vasculature in transplants. It is characterized by a concentric vascular intimal lesion comprised of smooth muscle-like cells (SMLC) and abnormally laid extracellular matrix and may simply be considered a result of abnormal stereotypic healing following alloimmune induced vascular injury [15,94].

Numerous studies show that macrophages are associated with the development of GVD; these cells have been observed in the lesions of GVD [95-99]. Macrophage depletion, but not inhibition of their ability to phagocytose, suppressed the development of cardiac graft vascular disease [100], suggesting that macrophages likely promote GVD through the production of proinflammatory, cytotoxic and trophic mediators but not their function as antigen-presenting cells. A study in kidney transplants showed that treatment with a macrophage inhibitor prevented progressive glomerulosclerosis, interstitial fibrosis, and arterial obliteration [101]. A more recent clinical study revealed that in heart transplants with very late rejection (> 7 years following transplantation), the presence of intravascular macrophages and donor specific antibodies are robust predictors of the development of more severe GVD [102]. Therapies effective in reducing GVD have also been shown to be associated with a significantly deceased macrophage infiltration [103,104]. These recent studies further confirmed that macrophages play a role in the pathogenesis of GVD.

Numerous mechanisms have been identified by which macrophages may promote the development of GVD. They may act as the predominant effector cells in CD4^+^ T cell-mediated delayed type hypersensitivity and have been shown to induce tissue and vascular damage through the production of eicosanoids, deleterious proteases, ROS and nitric oxide [15]. Macrophages may also promote GVD through the production of proinflammatory cytokines including IL-1, TNF-α, IFN-γ, platelet-derived growth factor (PDGF) and transforming growth factor (TGF)-β [15] (Figure 1). Double knockouts of both TNF-α receptor-1 and -2 in the graft significantly attenuated GVD in heart transplants [105], suggesting that TNF-α mediated signaling also contributes to the development of GVD. IFN-γ is also an important cytokine in the development of chronic rejection. In a heart transplant model, IFN-γ was shown to be both necessary and sufficient to drive the development of GVD [106]. Following IFN-γ stimulation, it has also been shown that macrophages produce IL-12 and IL-18, which further activates CD4^+^ T cell production of IFN-γ, thus forming a positive feedback loop [107]. Additionally, SMLCs also produce IFN-γ following IL-12 and IL-18 stimulation [108], demonstrating that macrophages and SMLCs may work together to promote the development of GVD.

SMLCs that display a synthetic phenotype are the primary cells that populate the lesions of GVD [15]. SMLCs with both donor and recipient origins have been described [109,110]. Numerous chemokine receptors including CXCR3, CXCR4, CCR1, CCR2, CCR3 and CCR5 are expressed on SMLCs [111-114]. Thus, macrophages producing cognate chemokines may promote recruitment and retention of recipient derived SMLCs, which may then facilitate neointimal formation and the development of GVD [15,94,115]. In an endothelial injury model of fulminant pulmonary arterial hypertension, our group showed that macrophages are the prominent producers of LTB_4_ (described above) and, in addition to causing EC apoptosis, also promote vascular smooth muscle cell proliferation and vascular remodeling. Blockade of LTB_4_ production by inhibition of the enzyme LTA_4_ hydrolase efficiently reopens obstructed pulmonary arterioles and reverses severe pulmonary arterial hypertension [77]. Together, these studies suggested that blockade of the chemokine signaling involved in macrophage recruitment and its production of the proinflammatory mediator may prevent/reverse GVD.

Of note, despite increasing appreciation that microvascular loss in solid organ transplants may play a causal role in the development of graft fibrosis and chronic rejection [19,30,69,71-74], GVD does not describe the pathology of the capillary loss and subsequent abnormal angiogenesis (e.g. microvascular loss in the airway transplant undergoing rejection as we described [19]). Interestingly, we recently found that macrophage infiltration is nearly absent around the remodeled capillaries of the chronically rejected airway transplants (unpublished observation). It is therefore possible that macrophages may only have significant effects on the microvasculature during IRI and AR.

## Concluding remarks

Macrophages, historically thought of as ‘accessory cells’ with a poorly-described secondary function, are now emerging as an important cell type in solid organ transplantation. Compelling preclinical and clinical studies have shown that macrophages not only promote graft injury and GVD, but also participate in tissue repair, including microvascular repair, in different types of transplant related injury. Strategies for macrophage-centered therapeutics may include macrophage depletion or polarization to a reparative phenotype. Depletion may be achieved by direct killing through antagonism of CSF-1R or CSF-1 [85] or by blockade of recruitment by targeting CCR and CXCR mediated chemotactic pathways [57-60,116-118]. In transplantation rejection, when injurious and reparative classes coexist within the allograft, polarization of macrophages to a reparative phenotype may be a better strategy. Indeed, commonly used immunosuppressive drugs, such as glucocorticoids and mammalian target of rapamycin (mTOR) inhibitors, in addition to antagonizing T cells, are known to polarize macrophages to a suppressive phenotype [119,120]. More recently, *ex vivo* expanded regulatory macrophages were characterized and used in clinical trials, which may represent a promising therapeutic modality to prolong graft survival [92].

In summary, macrophages play numerous roles in solid organ transplant injury and rejection. A better understanding of how macrophages both damage and repair the allograft circulatory system in different types of transplant injury and rejection is required to further promote this cell, in all its myriad manifestations, as a promising therapeutic target. With advances in the fields of genomic analysis and systems biology, an improving delineation of macrophage subtypes is already occurring and opening new doors of investigation.

## Abbreviations

AAM: Alternatively-activated macrophage; AR: Acute rejection; BLT1: Leukotriene B_4_ receptor type-1; CAM: Classically-activated macrophage; CCR: CC chemokine receptor; CSF-1: Colony stimulating factor-1; CXCR: CXC chemokine receptor; CX3CR: CX3C chemokine receptor; DSA: Donor specific antibody; EC: Endothelial cell; FGF: Fibroblast growth factor; GVD: Graft vascular disease; HIF: Hypoxia inducible factor; HMGB-1: High-mobility group box-1; IDO: Indoleaminepyrrole 2,3-dioxygenase; IFN: Interferon; IL: Interleukin; iNOS: Inducible nitric oxide synthase; IRI: Ischemia reperfusion injury; LPS: Lipopolysaccharide; LTB4: Leukotriene B_4_; MCP-1: Monocyte chemoattractant protein-1; M-CSF: Macrophage-colony stimulating factor; MIF: Migration inhibitory factor; Mreg: Regulatory macrophage; mTOR: Mammalian target of rapamycin; NF-κB: Nuclear factor κB; PAF: Platelet activating factor; PDGF: Platelet-derived growth factor; PLGF: Placental growth factor; RNS: Reactive nitrogen species; ROS: Reactive oxygen species; SDF: Stromal cell-derived factor; SK-2: Sphingosine kinase-2; SMLCs: Smooth muscle-like cells; TGF: Transforming growth factor; TLR: Toll-like receptor; TNF: Tumor necrosis factor; VHL: Von Hippel–Lindau; VEGF: Vascular endothelial growth factor.

## Competing interests

The authors declare that they have no competing interests.

## Authors’ contributions

XJ, WT, YS, JQ and MN prepared and wrote the manuscript. All authors read and approved the final manuscript.
